# Deracemization by coupling electrochemically assisted racemization and asymmetric crystallization

**DOI:** 10.1039/d5cc05874k

**Published:** 2025-10-27

**Authors:** Anne-Sophie Léonard, Morgan Regnier, Susanna Bertuletti, Sjoerd W. van Dongen, Roberta Listro, Michel Leeman, Richard M. Kellogg, Timothy Noël, Willem L. Noorduin

**Affiliations:** a AMOLF Science Park 104 1098 XG Amsterdam The Netherlands; b Van’t Hoff Institute for Molecular Sciences, University of Amsterdam Science Park 904 Amsterdam The Netherlands; c Symeres Kadijk 3 Groningen 9747 AT The Netherlands; d Department of Drug Sciences, University of Pavia 27100 Pavia Italy; e Kellogg Beheer B. V. Zernikepark 12, Unit 1.31 9747 AN Groningen The Netherlands

## Abstract

Amino acid derivatives of *tert*-leucine and phenyl glycine along with plant growth retardant and the precursor of fungicide paclobutrazol are deracemized by combining *in situ* electrochemical base generation to induce racemization and crystallization-induced chiral amplification in a one-pot, two-step deracemization procedure. Full enantioselective conversion (e.e. > 99%) of a mixture of enantiomers towards the desired handedness is achieved.

## Abstract 2

Amino acid derivatives and paclobutrazol precursor are deracemized by combining *in situ* electrochemical base generation, inducing racemization and crystallization-driven chiral amplification in a one-pot, two-step approach.

## Graphical abstract

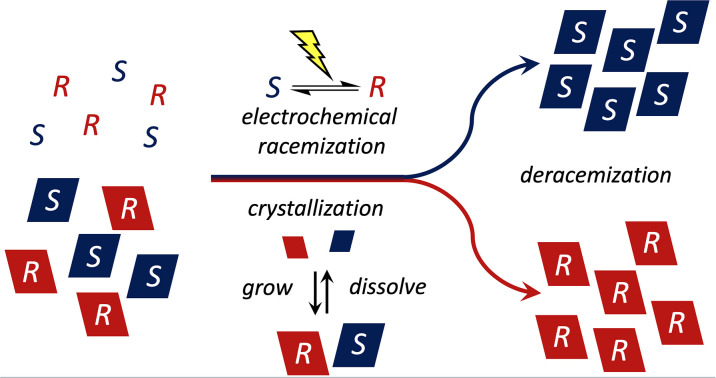

Enantiomerically pure compounds are crucial in the synthesis of bioactive molecules such as pharmaceuticals and agrochemicals.^1^ To obtain compounds in their desired enantiomeric form, chiral resolution techniques like diastereomeric salt formation, kinetic resolution, and chiral chromatography are used extensively.^2^ However, these separation methods intrinsically suffer from limited efficiency when the undesired enantiomer cannot be recycled.

To overcome this inefficiency, asymmetric synthesis and catalytic deracemization—where racemic mixtures are converted into the desired enantiomer—have been widely explored.^3^ Also, electrochemical deracemization and asymmetric synthesis have gained increasing interest due to their sustainability, cost-effectiveness, and high regio- and chemoselectivity.^4^ Despite recent progress, significant challenges remain in balancing electrochemical reactivity and enantioselectivity. Moreover, the need for highly effective chiral catalysts and auxiliaries that can precisely direct the reaction toward the desired enantiomer has so far hindered the widespread adoption of electrochemical deracemizations.^5^

Crystallization-induced deracemization can offer a solution to the challenges of using complex chiral catalysts and workflows. In crystallization-induced deracemizations, scalemic mixtures of homochiral crystals (*i.e* conglomerates) continuously grow and dissolve in a slurry, while a solution-phase racemization reaction enables the conversion of the minor enantiomer into the desired majority enantiomer.^6,7^ In contrast to typical asymmetric catalysis processes and deracemization reactions, a key advantage of this approach is that the crystals only incorporate one of the two enantiomers: they are intrinsically enantioselective. Hence, no complex chiral catalyst is required, and the product can amplify its own formation. Because of this inherently autocatalytic behaviour, the deracemization process continues until all crystals of the undesired enantiomer are fully converted into crystals of the desired enantiomer of choice, such that virtually enantiomerically pure products can be obtained in near quantitative yields.

Crystallization-induced deracemization has been successfully applied to various classes of molecules and using diverse crystallization methods.^8–16^ However, less attention has been dedicated to the study and development of racemization methods. Motivated by the promising potential of electrochemical reactions, we aim to explore their implementation in crystallization-induced deracemization processes. Although one example of electrochemical racemization has been disclosed in a patent application,^17^ the use of electrochemistry for racemization processes remains underexplored, let alone in tandem with crystallization-induced chiral amplification processes.

To realize such an electrochemically coupled deracemization process, a key challenge lies in integrating reaction conditions that facilitate electrochemically assisted racemization with continuous crystallization.^15^ To achieve this aim, it is essential that the electrochemically assisted racemization reaction is: (i) fast and clean, to allow deracemization on practical timescales while preventing undesired side reactions or degradation of the target molecule; and (ii) compatible with the crystallization-induced deracemization process, such that the electrochemistry and growth/dissolution of crystals do not impede each other.

To satisfy these essential prerequisites, we here propose to generate electrochemically a methoxide base that promotes solution phase racemization of versatile building blocks such as chiral amino acid derivatives and other bioactive compounds for a crystallization-induced deracemization process (Fig. 1). The key idea is that our strategy overcomes the problem of compatibility between racemization reaction and the crystallization process: (i) the electrogenerated methoxide base enables fast and clean racemization of Schiff base amino acid derivatives and the paclobutrazol precursor; (ii) methanol acts both as precursor of the electrochemically generated catalyst and as solvent for the crystallization. In that way, both electrogeneration and crystallization are possible in a one-pot, two-step approach by simply removing the electrodes after electrogeneration of the methoxide base to perform crystallization-induced chiral amplification using attrition.

**Fig. 1:**
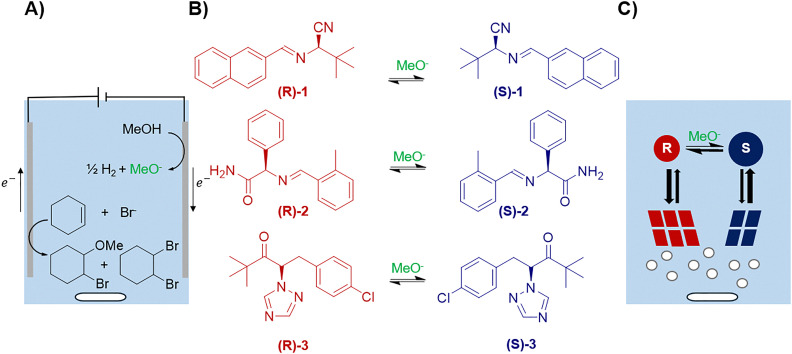
Electrochemically assisted racemization in a solution of Et_4_NBr for the crystallization-induced deracemization. (A) Electrogeneration of a methoxide base as racemization catalyst. (B) Derivatives of *Tert*-leucine 1, phenyl glycine 2, and ClTAK 3 undergo racemization by electrogenerated methoxide. (C) After generation of the methoxide base, a slurry is created and solution phase racemization induces deracemization in the solid phase during crystallization under attrition using glass beads.

We here demonstrate the proof-of-principle of deracemization *via* electrochemically assisted racemization for derivatives of amino acids *tert*-leucine (1), phenyl glycine (2) and plant growth retardant and fungicide paclobutrazol precursor CITAK (3) (Fig. 1). These derivatives are ideal for demonstrating the proof-of-concept as 1, 2 and 3 crystallize as conglomerates, undergo racemization in the presence of a base, and have been successfully deracemized by attrition and temperature cycling.^7,9,12^ Therefore, we suggest that electrochemically generating the base *in situ* could be of interest for an easier and cleaner procedure.

For the electrogeneration of the racemization catalyst, we drew inspiration from previous work that has shown that methoxide base can form through the electrochemical reduction of methanol.^18^ However, it was unclear which oxidative counterreaction was suitable for our specific racemization reaction, and how this electrochemically assisted racemization reaction could be combined with crystallization conditions. To this aim, we identified suitable conditions for the electrochemically assisted racemization and then used the optimized conditions for crystallization-induced deracemization.

We first investigated the racemization of (*R*)-1 using electrochemically generated methoxide base (Fig. 2). Initially, we hypothesized that oxidation of the bromide from tetraethylammonium bromide (Et_4_NBr) could be sufficient to conduct the reaction, but no racemization was observed under these conditions (see SI). To overcome this problem, we screened additives (see SI) to find a suitable counter-reaction. We identified cyclohexene as a suitable substrate for the oxidative counterreaction to produce hydrogen and the methoxide base. Based on previous work on halide-mediated electrochemical oxidation of alkenes,^19^ we anticipated the *in situ* generated bromine from the supporting electrolyte to obtain the oxidized cyclohexene products: 2-dibromocyclohexane and 1-bromo-2-methoxycyclohexane (Fig. 1A).

**Fig. 2:**
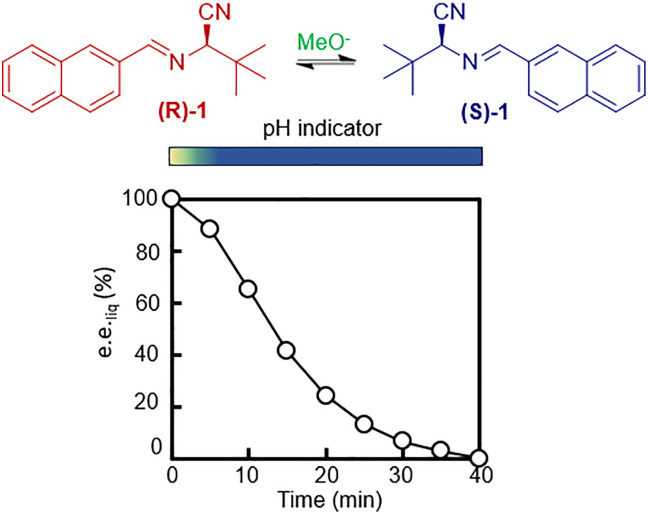
Solution phase racemization of (1) using electrogenerated methoxide base (A). The enantiomeric excess in solution (e.e._liq_) decreases over time after an initial drop in pH. The line is a guide to the eye.

To perform this electrochemically assisted racemization, we prepared an undersaturated solution of (*R*)-1 in methanol together with cyclohexene and Et_4_NBr. We inserted graphite electrodes in the resulting clear liquid and applied constant potential to initiate the reaction. We followed the racemization in time. To this aim, we took samples every 5 minutes, quenched the racemization by adding NH_4_Cl and analysed the enantiomeric excess of the liquid (e.e._liq_) using chiral high performance liquid chromatography (HPLC, see SI).

We observed that (*R*)-1 racemizes within 40 minutes e.e._liq_ > 98% to e.e._liq_ < 2% (Fig. 2). For typical racemization reactions, where the racemization catalyst is present from the start, we expect pseudo-first order reaction kinetics such that e.e._liq_ decreases exponentially in time.^8^ In contrast, we here found that initially racemization was slow, and only after 6 minutes the e.e._liq_ started to decrease exponentially. This slow start of the racemization is consistent with the time required for the electrochemical generation of sufficient amounts of methoxide base used as racemization catalyst. Indeed, following the pH over time using wide range indicator solution (pH 4–12), showed that only after the induction time (*ca.* 6 minutes), the reaction mixture was sufficiently basic (pH 10) to enable effective racemization (see SI). Importantly, quantitative HPLC showed no evidence of decomposition of 1, hence demonstrating that electrochemically assisted racemization is applicable on practical time scales. We further investigated the mechanism of the electrochemical generation of the racemization catalyst. Consistent with the proposed reaction (Fig. 1A), ^1^H-NMR confirmed that the double bond of cyclohexene is reduced during the process. Moreover, the reaction products 1,2-dibromocyclohexane and 1-bromo-2-methoxycyclohexane were detected by gas chromatography-mass spectroscopy (GC-MS, SI) while hydrogen production was confirmed by gas chromatography with a thermal conductivity detector (GC-TCD, SI). Consistent with the proposed mechanism, racemization of 1 did not take place without cyclohexene but instead degradation of 1 was observed (SI). We also found that racemization was not possible using aromatic compounds such as toluene or saturated alkanes such as cyclohexane as precursor for the oxidative counterreaction, which is also consistent with the mechanism (SI). As expected, racemization was possible using other non-conjugated alkenes, such as limonene, linalool and myrcene (SI). Cyclohexene is the preferred non-conjugated alkene as it is cost-effective.

Having established suitable conditions for the electrogeneration of the racemization catalyst, we integrated the electrochemical assisted racemization with a crystallization process to achieve the deracemization of 1. To this aim, we first electrochemically induced racemization in an undersaturated solution of (*R*)-1 in methanol together with cyclohexene and Et_4_NBr. Once electrochemical assisted racemization was observed with HPLC, the electrodes were removed and (*RS*)-1 crystals together with seeds of the desired enantiomer (*R*)-1 were added to create a slurry with an enantiomeric excess in the solid phase e.e._solid_ = *ca.* 20%. Glass beads were added to this slurry, and the vial was placed in a thermostatically controlled ultrasonic bath to induce attrition of the crystals, effecting continuous growth and dissolution. Within 24 hours, seeding with (*R*)-1 (e.e._solid_ = *ca.* 20%) resulted in complete deracemization of the solid phase into (*R*)-1 (Fig. 3A). Likewise, seeding the slurry with (*S*)-1 (e.e._solid_ = *ca.* 20%) resulted into complete conversion of the solid phase into (*S*)-1 (Fig. 3B), which demonstrates directed control of the deracemization towards a chiral configuration of choice.

**Fig. 3:**
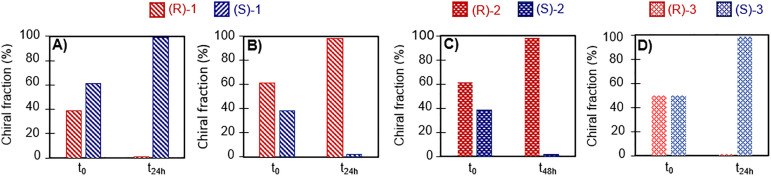
Solid phase deracemization by combining electrochemically induced liquid phase racemization and attrition, showing complete deracemization to (A) (*R*)-1, (B) (*S*)-1, (C) (*R*)-2, and (D) (*R*)-3.

To demonstrate the generality of our approach, we investigated compound 2 which has been previously deracemized using crystallization induced deracemization under based-catalysed racemization.^7^ Following a procedure akin to 1 (see SI), we first electrochemically generated the methoxide base to induce racemization of 2. The full racemization of 2 (70 minutes) was slower than 1 (40 minutes). This difference in racemization rate is expected: the amide group in 2 is less electron withdrawing than the nitrile group in 1, such that 2 racemizes slower than 1. As consequence of the slower racemization reaction of 2, we found that the deracemization is also slower (starting with e.e._solid_ = *ca.* 20%, 48 hours for 2 instead of 24 hours for 1). Nevertheless, we could still achieve complete conversion in the solid phase within 48 hours to either enantiomer by seeding (*S*)-2 or (*R*)-2 (Fig. 3C). As expected, lowering the initial enantioenrichment to e.e._solid_ = 10% and even 0% still resulted in deracemization (72 hours and 7 days respectively, see SI).

To further establish the generality of this electrochemically coupled deracemization process, we also investigated the deracemization of compound 3. CITAK (3) has previously been deracemized using sodium hydroxide as racemizing agent.^9^ Using our electrochemical method (see SI), 3 racemized in 20 min, which is substantially faster than compound 1 and 2. This can be explained by the greater electron withdrawing ability of the triazole group around the chiral center. Even without adding any initial enantioenrichment, full deracemization of 3 was obtained within 24 hours (Fig. 3D).

Here we have introduced an electrochemically assisted racemization for complete crystallization-induced chiral amplification. The key idea of our strategy is that the electrochemically assisted racemization conditions are compatible with the crystallization induced deracemization such that coupling of both processes is possible. We foresee that this electrochemically assisted deracemization approach can directly be extended to other relevant chiral molecules such as precursors for naproxen, levetiracetam and brivaracetam.^11,13^ Instead of inducing growth/dissolution by grinding crystals, deracemization may also be achieved by processes such as temperature cycling or solvent cycling.^14,16^ Furthermore, preliminary studies (see SI) show that the racemization can be performed under flow conditions, making it feasible to operate the electrochemically assisted deracemization in a continuous flow setup for fast and scalable production of enantiomerically pure molecules.^20^

Moreover, we foresee that electrochemical racemization may be ideal for optimizing the rate of crystallization-induced deracemization, by selectively switching the racemization reaction on and off during growth and dissolution steps respectively.^21^ Finally, hydrogen transfer reactions may also enable electrochemically assisted racemization of the here-presented substrates, while oxidation and reduction cycles may potentially expand the scope of deracemizable molecules.^17,22^

We thank the Dutch Research Council (NWO), AMOLF funds for Topsector-related research and European Union H2020 research and innovation program program for supporting our research under the Marie Skłodowska-Curie Grant Agreement.

## Conflicts of interest

The authors declare no conflict of interest.

## Supplementary Material

CC-061-D5CC05874K-s001

## Data Availability

The data supporting this article have been included as part of the supplementary information (SI). Supplementary information is available. See DOI: https://doi.org/10.1039/d5cc05874k.
