# Supplementary material for: Deracemization by coupling electrochemically assisted racemization and asymmetric crystallization
Source: Chem Commun (Camb). 2025 Oct 27;61(95):18834–7. doi: 10.1039/d5cc05874k (PMC12580979; doi:10.1039/d5cc05874k)
Supplement: CC-061-D5CC05874K-s001 [file CC-061-D5CC05874K-s001.pdf]

Supplementary Information (SI) for ChemComm.
This journal is © The Royal Society of Chemistry 2025

Supporting information

Deracemization by Coupling Electrochemically Assisted Racemization and Asymmetric Crystallization

Anne-Sophie Léonard[†a], Morgan Regnier[†b], Susanna Bertuletti[a], Sjoerd W. van Dongen[a], Roberta Listro[c,d], Michel Leeman[c], Richard M. Kellogg[e], Timothy Noël[b], Willem L. Noorduin*[a,b]

[a]AMOLF, Science Park 104, Amsterdam 1098 XG, The Netherlands.
[b]Van't Hoff Institute for Molecular Sciences, University of Amsterdam, Science Park 904, Amsterdam, The Netherlands
[c]Symeres, Kadijk 3, Groningen 9747 AT, The Netherlands
[d] University of Pavia, Department of Drug Sciences, 27100, Pavia, Italy
[e] Kellogg Beheer B.V., Zernikepark 12, Unit 1.31, 9747 AN Groningen, The Netherlands

[†]These authors contributed equally.
*Corresponding author: noorduin@amolf.nl

Table of Contents

1. General information 3

2. Synthesis, HPLC analyses and quantitative method 4

Synthesis of compound tert-leucine 4

Synthesis of compound phenyl glycine 4

Synthesis of compound CITAK 4

HPLC methods 5

Quantitative method 5

3. Batch electrochemical cell design 6

4. Flow electrochemical reactor design 6

5. General procedures 6

Electrochemically assisted racemization 6

HPLC analyses for racemization 7

Kinetic measurement for racemization 9

Crystallization induced deracemization 9

HPLC analyses deracemization 9

6. Electrochemically assisted racemization optimization 11

Control experiments 13

7. Mechanistic investigation 14

Hydrogen headspace measurement 14

Additive degradation 15

GC-MS of the reaction mixture 15

Cyclic voltammetry study 16

pH control test 16

8. Determination of solubility via Crystal 16 17

9. ^{1}H NMR 17

10. Flow chemistry 19

11. References 19

1. General information

Reagents and consumables. All reagents and solvents were bought from Sigma Aldrich, TCI Europe N.V., Fluorochem, VWR International and Biosolve and used as received. Disposable syringes were bought from BD Discardit II®, NORM-JECT® purchased from VWR Scientific. High grade HPLC solvent (≥ 99%) were purchased from VWR chemicals. Filter paper 47 mm (VWR Internationals), 0.2 µm PTFE syringe filter (VWR Internationals), 1mL syringe (VWR Internationals) and 2 mL HPLC vials (29651-U Supelco, Merck) were used to prepare the HPLC sample. A wide range 4 pH indicator solution from HACH was used.

Electrochemistry. Graphite SK-50 electrodes were purchased from IKA. Batch electrolysis was performed using the IKA Electrasyn 2.0. Cyclic voltammograms were recorded with a potentiostat Emstat3 + (PalmSens BV), and a 3-electrodes cell equipped with a 3 mm diameter glassy carbon disk working electrode a, a platinum wire counter electrode and a silver wire in a solution of blank electrolyte (0.1 M Et_4NBr/MeOH) as pseudo-reference. Subsequently, ferrocene was added at the end of the experiment and a CV recorded to get the potential of the Fc/Fc^+ redox couple. The measurements were performed in dry 0.1M Et_4NBr/MeOH (5 mL) solutions purged of air with MeOH-saturated nitrogen. Unless specified, a scan rate of 100 $mV{\cdot}s^{-1}$ was used. Polishing of the electrodes after batch electrolysis and cyclic voltammetry was performed using 1, 0.3 and 0.05 µm alumina powder from CH instruments, Inc.

NMR spectroscopy. ^1H (300 MHz) spectra were recorded at ambient temperature using a Bruker AV400. ^1H NMR spectra are reported in parts per million (ppm). The multiplicities of signals are designated by the following abbreviations: s (singlet), d (doublet), q (quartet), m (multiplet), dd (doublet of doublets). NMR data was processed using the MestReNova 14 software package. Known products obtained were characterized by comparing to the corresponding ^1H NMR from the literature.

Gas chromatography. Analyses were performed on a Shimadzu Nexis GC-2030 gas chromatograph, equipped with a thermal conductivity detector (TCD). A two column system (SH-Q-BOND and SH-Msieve 5A) with a bypass was employed to analyze all relevant gaseous compounds. When starting the analysis, 50 µL (sample loop volume) of the gaseous sample is taken and sent to the column. The method uses a split injection (split ratio 5.0) at the temperature of 80 °C. A helium carrier gas is used, and all compounds pass the first column (QBOND). Next, the light gases are parked in the second column (Msieve 5A) and after the methane exits the first column the valve switches and the carbon dioxide will bypass the second column to go to the TCD (200 °C, helium reference) and FID detector (jetanizer, 280 °C), as shown in Figure S3. The bypass time is of 3.45 minutes. After the carbon dioxide has passed the FID, the gases are flowed to the detectors. The retention times of methane and carbon dioxide need to be separately measured to determine the time of the valve switches and develop the method. During the analysis the oven is kept at 40 °C. GC-MS data was gathered using an Agilent 5977C GC/MSD System.

High pressure liquid chromatography analyses. Chiral-phase HPLC analyses were performed using a Jasco LC-4000 series HPLC equipped with CHIRALPAK OJH column (250 x 4.6 mm, 5 µm) or CHIRALPAK ODH column (250 x 4.6 mm, 5 µm) and Agilent Technologies Infinity 1260 HPLC system equipped with CHIRALPAK IA column (250 x 4.6 mm, 5 µm).

Deracemization setup. Sonication bath (Ultrasonic Cleaner USC-THD, VWR internationals) was connected to a cooler system Julabo FE500 15 °C. Sampler holder and closing was created by AMOLF's workshop department.

Crystal 16. Solubility analyses for derivatives of tert-leucine (**1**), phenyl glycine (**2**) and CITAK (**3**) were performed with a Crystal 16 from Technobis using a rate of 0.5 °C/min and under 1000 rpm.

2. Synthesis, HPLC analyses and quantitative method

Synthesis of compound tert-leucine

(E)-3,3-dimethyl-2-((naphthalen-2-ylmethylene)amino)butanenitrile (tert-leucine, **1**) was synthesized by Symeres according to the reported procedure.[1]

Synthesis of compound phenyl glycine

(E)-2-(benzylideneamino)-2-phenylacetamide was synthesized as followed (phenyl glycine, **2**) starting from commercially available (RS)-phenylglycine ((RS)-Phg) and its subsequent acid hydrolysis.[2]

Step 1: The resulting solution of (RS)-Phg (30,3 g, 0.20 mol, 1 equiv) in 200 mL of methanol was cooled down to 0°C. Thionyl chloride (28.5 g, 0.24 mol, 1 equiv) was slowly introduced over several hours, ensuring the temperature did not exceed 20 °C. The reaction mixture, a clear solution, was stirred at room temperature for 18 hours, then heated under reflux for one hour to expel sulfur dioxide. The solvent was then reduced under vacuum to about 60 mL. To induce crystallization of the (RS)-Phg methyl ester hydrochloride salt, 300 mL of methyl tert-butyl ether (MTBE) was added. The resulting crystals were filtered and vacuum-dried.

Step 2: The aforementioned crystals were gradually added to a stirred solution of 150 mL concentrated ammonia. During the addition, the (RS)-Phg amide began to precipitate. Stirring continued for several hours to ensure full conversion of the ester (monitored via TLC using a solvent system of chloroform, methanol, and concentrated ammonia in a 60:45:20 ratio). The amide was collected by filtration, rinsed with cold water (noting a solubility of approximately 5 wt%), and dried. The process yielded around 75% of the desired (RS)-phenylglycine amide.

Step 3:
To a stirred mixture of (RS)-Phg amide (22.5 g, 0.15 mol, 1 equiv) in 75 mL water and 150 mL methanol at room temperature, 2-methylbenzaldehyde (18,0 g, 0.15 mol, 1 equiv) was added over one hour. After approximately 20% of the aldehyde was added, crystallization often began on its own; otherwise, seeding with a small amount of racemic product helped initiate the process. The mixture, now a thick suspension, was stirred for 20 hours, filtered, and the solid washed with a 2:1 methanol/water solution (50 mL) followed by 100 mL of MTBE. The crystals were dried under reduced pressure, yielding 35 g (95%) of (RS)-**2** as white crystals.

Synthesis of compound CITAK

1-(4-chlorophenyl)-4,4-dimethyl-2-(1H-1,2,4-triazol-1-yl) pentan-3-one (CITAK, **3**) was synthesized inspired by a reported procedure in a two steps approach.[3]

Step 1: A mixture 1,2,4-triazole (4.97 g, 0.07 mol, 1 equiv), chloropinacolone (10.7 g, 0.08 mol, 1.1 equiv), and potassium carbonate (14.9 g, 0.11 mol, 1.5 equiv) was prepared in 100 mL of acetonitrile. The reaction was heated under reflux and stirred for 15 hours. Upon completion, the mixture was filtered and the solvent was evaporated under reduced pressure. The resulting residue was treated with water and extracted three times with ethyl acetate. The combined organic extracts were dried over anhydrous magnesium sulfate, filtered, and concentrated under vacuum. The product was further purified by washing it with cold diethyl ether. This process yielded 10.9 g of product (91%).

Step 2: To a stirred suspension of sodium hydride (0.28 g, 0.012 mol, 1 equiv) in 3.5 mL of DMF, a solution of TP (2.0 g, 0.012 mol, 1 equiv) dissolved in 3,5 mL of DMF was added slowly, dropwise, at 0 °C. After the addition was complete, chlorobenzyl chloride (2.12 g, 0.013 mol, 1.1 equiv) was introduced at the same temperature. The reaction mixture was then allowed to warm to ambient temperature and stirred continuously for 8 hours and 30 minutes and quenched with water when completed. Subsequently, the mixture underwent liquid-liquid extraction with ethyl acetate, performed three times. The combined organic extracts were dried over anhydrous magnesium sulfate, filtered, and concentrated under reduced pressure to eliminate residual DMF. The resulting crude product was purified by crystallization from hexane over a period of at least 24 hours, yielding 2.66 g (76%) of ClTAK as a white solid.

HPLC methods

Tert-leucine (**1**) was analyzed on a CHIRALPAK IA column (250 x 4.6 mm, 5 µm) using n-heptane and 1-propanol in a 95:5 ratio with a flow of 0.7 mL/min. The injection volume was 4 µL and the UV detector wavelength was 220 nm. Typical retention time for was 5.17 min for anisole (external standard), 7.90 min for (S)-**1** and 9.05 min (R)-**1**.

Phenyl glycine (**2**) was analyzed on a CHIRALPAK OJ-H column (250 x 4.6 mm, 5 µm) using n-heptane and ethanol in a 40:60 ratio flow. The injection volume was 2 µL and the UV detector wavelength was 240 nm. Typical retention time was 9.18 min for anisole (external standard), 11.59 min for (S)-**2** and 16.01 min (R)-**2**.

CITAK (**3**) was analyzed on a CHIRALPAK OD-H column (250 x 4.6 mm, 5 µm) using n-heptane and 1-propanol in a 80:20 ratio with a flow of 1 mL/min. The injection volume was 4 µL and the UV detector wavelength was 220 nm. Typical retention time for was 4.2 min for anisole (external standard), 7.1 min for (R)-**3** and 8.4 min (S)-**3**.

Quantitative method

Anisole 0.2% was added to the HPLC vial as external standard (ES) to evaluate the concentration of R and S.

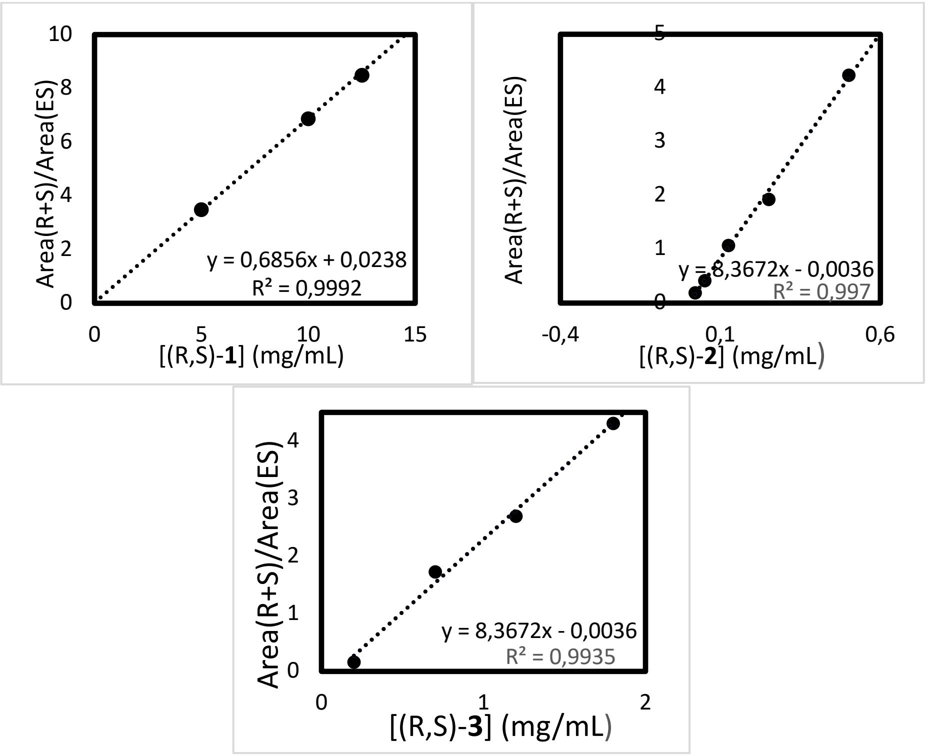

Figure S1: Quantitative curve for **1**, **2** and **3** using anisole.

3. Batch electrochemical cell design

Batch experiments were conducted in an undivided cell of 10 mL, equipped with 2 graphite electrodes (53x8x2 mm). In each experiment, the electrodes were immersed in 7.5 mL of reaction media corresponding to a total effective surface of 1.5 cm^2.

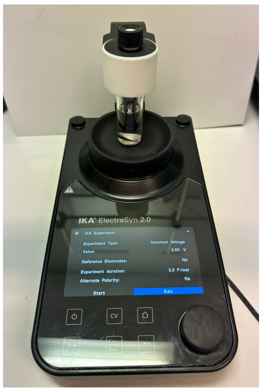

Figure S2: Electrasyn 2.0 undivided cell used for batch electrochemical reactions.

4. Flow electrochemical reactor design

For continuous-flow experiments, an electrochemical microflow reactor developed by Noël's group was used.[4] This reactor contains two graphite electrodes (120x55x2 mm), separated by a PTFE gasket of 250 µm thickness divided in 8 channels (106x3 mm per channels). With this configuration the reactor has as an active internal volume of 0.7 mL and an active surface of electrode of 25.5 cm^2. The assembly consists of stacking and screwing together PTFE and stainless-steel plates (Figure S3), the support is then connected to the power supply. For each reaction, one reactor volume was discarded before starting the collection.

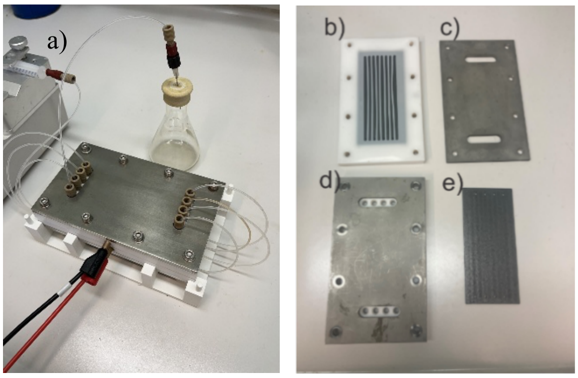

Figure S3: a) Flow electrochemical reactor assembled with a b) gasket of 250 µm; c) Stainless steel plate; d) PTFE plate topped by a stainless-steel plate; e) Graphite electrode.

5. General procedures

Electrochemically assisted racemization

To a 10 mL vial equipped with a stirring bar and 2 graphite electrodes, the substrate (0.2 mmol, 1 equiv), Et_4NBr (158 mg, 0.2 mmol, 1 equiv) and additive (0.2 mmol, 1 equiv) were added in 7.5 mL MeOH. The solution was stirred until homogeneous and electrolyzed for 2 h setting the cell potential to 2.0V. Then, 0.05 mL were sampled from the mixture and quenched in 0.15 mL of a NH_4Cl solution in EtOH (0.1 M). The final composition and enantiomeric excess in the liquid phase ($e.e._{liq}$) were determined by chiral-phase HPLC (see section 2 for methodology). Subsequently, the graphite SK-50 electrodes were washed with MeOH (3 × 20 mL) and polished using 1 and 0.3 µm alumina powder.

HPLC analyses for racemization

To follow the racemization kinetics, 0.05 mL of liquid phase reaction together with 0.15 mL of anisole 2% (external standard) was mixed with 0.15 mL of a NH_4Cl solution in EtOH (0.1 M) and 1.15 mL of MeOH to stop racemization. This sample was analyzed using chiral-phase HPLC using the methods described above. Full racemization of (S)-**1** and (R)-**1** were obtained after 40min (See HPLC section for (R)-**1**) while full racemization of (S)-**2** and (R)-**2** were obtained after 70 min (See HPLC Chromatograms for (S)-**2**) and full racemization of (S)-**3** and (R)-**3** were obtained after 20 min.

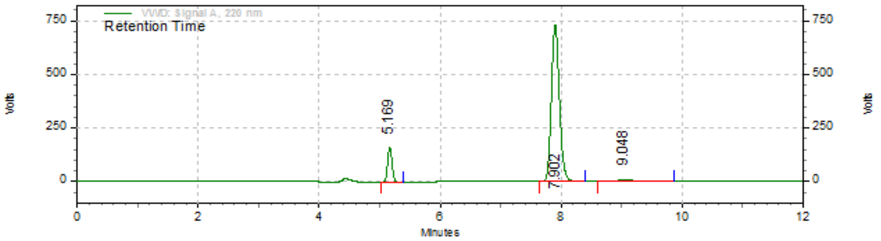

VWD: Signal A,
220 nm Results

Retention Time	Area	Area %	Height	Height %
5.169	14409488	11.04	2716279	17.95
7.902	113039378	86.64	12242429	80.88
9.048	3019137	2.31	177101	1.17

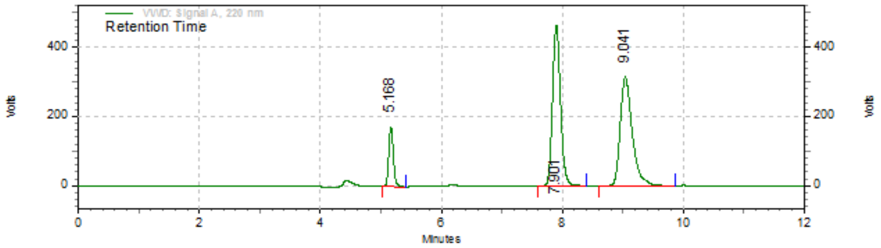

VWD: Signal A,
220 nm Results

Retention Time	Area	Area %	Height	Height %
5.168	15129848	9.49	2861019	17.97
7.901	71788852	45.02	7788259	48.91
9.041	72531947	45.49	5275408	33.13

Figure S4: Start (top) and end (bottom) of racemization for (S)-**1**.

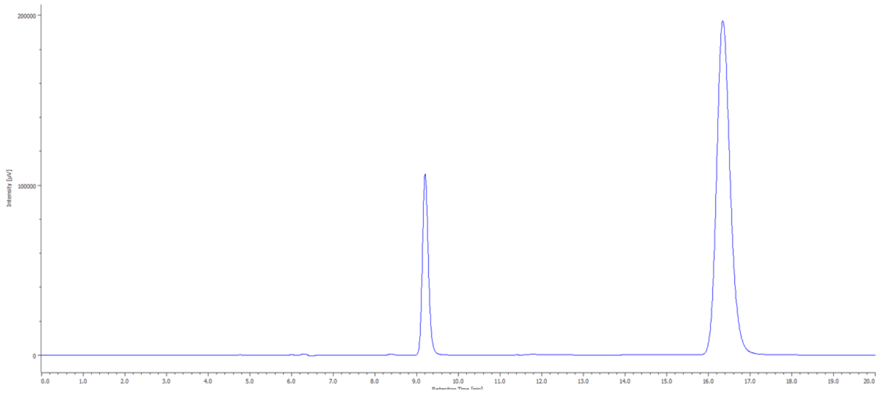

tR	Area	Height	Area%	Height%
9.200	1060695	106465	19.534	35.243
16.333	4369401	195624	80.466	64.757

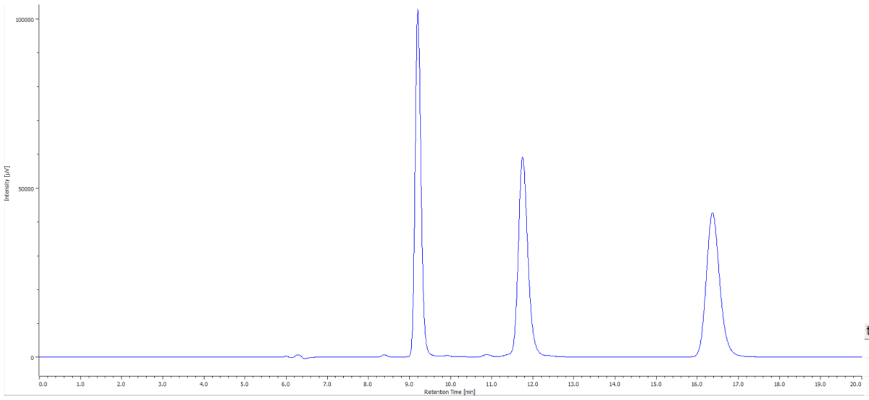

tR	Area	Height	Area%	Height%
9.200	1020417	102562	34.942	50.369
11.750	952761	58771	32.625	28.863
16.358	947150	42287	32.433	20.767

Figure S5: Start (top) and end (bottom) of racemization for (R)-**2**.

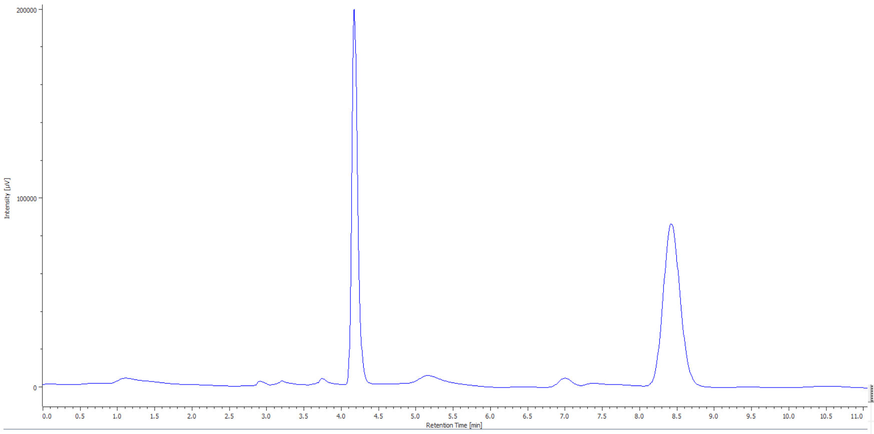

tR	Area	Height	Area%	Height%
4.175	1086827	197959	44.310	69.732
8.417	1365971	85927	55.690	30.268

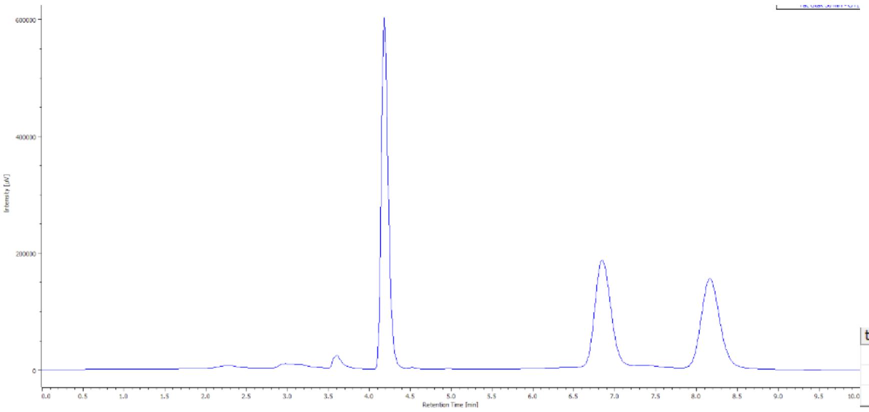

tR	Area	Height	Area%	Height%
4.183	3589561	600224	42.610	64.214
6.842	2431475	181709	28.863	19.440
8.158	2403245	152788	28.528	16.346

Figure S6: Start (top) and end (bottom) of racemization for (S)-**3**.

Kinetic measurement for racemization

Kinetic measurements have been performed on (R)-**1** according to the general procedure for racemization. 0.05 mL of the reaction mixture was taken every 5 minutes for 40 minutes and quenched with 0.150 mL of NH_4Cl solution in EtOH (0.1 M).

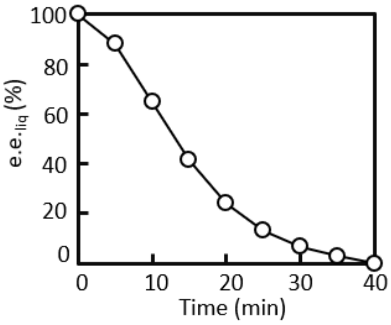

Figure S7: Racemization kinetic measurement of (R)-**1**. Line is a guide to the eye.

Crystallization induced deracemization

1mL of the resulting electrochemical racemization **1** mixture was transferred into a 2 mL vial together with 1 g of glass beads. 180 mg of (RS)-**1** and 30 mg seeds of pure R or pure S were added to the solution to obtain a slurry with 20% of enantiomeric excess in the solid phase ($e.e_{sol}$) (R or S). The vial was then placed on a sample holder and immersed into the sonication bath connected to a cooling system at 15 °C. The deracemization process took place within 24 hours.

7.5 mL of the resulting electrochemical racemization **2** mixture was transferred into a 20 mL together with 7.5 g of glass beads. 585 mg of (RS)-**2** and 90 mg seeds of pure R or pure S were added to the solution to obtain a slurry with 20% $e.e._{sol}$ (R or S). The vial was then placed on a sample holder and immersed into the sonication bath connected to a cooling system at 15 °C. The deracemization process takes place within 48 hours. Lowering the ee to 10% and 0% lead to deracemization time of 72 hours and about a week.

HPLC analyses deracemization

Sampling of the solid phase: 100-200 µL of the slurry was filtered and the resulting solid was dried. About 0.5 -1 mg is then added to 1.5 mL of MeOH and analyzed by HPLC

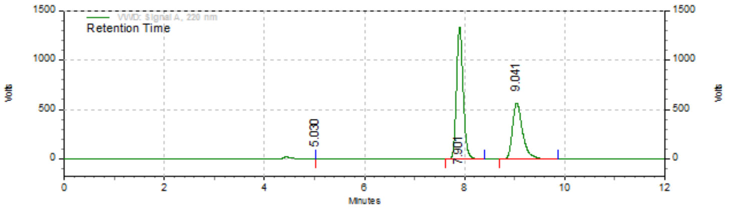

VWD: Signal A, 220 nm Results

Retention Time	Area	Area %	Height	Height %
5.030	0	0.00	0	0.00
7.901	206430738	61.04	22362288	70.18
9.041	131770867	38.96	9502488	29.82

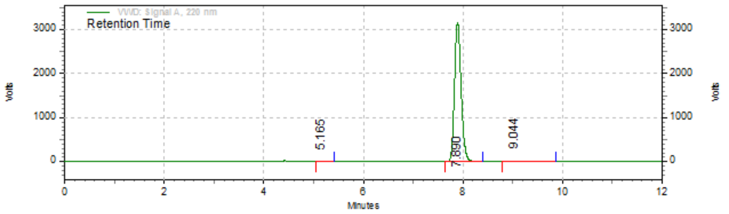

VWD: Signal A, 220 nm Results

Retention Time	Area	Area %	Height	Height %
5.165	164220	0.03	19987	0.04
7.890	491884916	99.05	53012560	99.48
9.044	4573737	0.92	258292	0.48

Figure S8: Start (top) and end (bottom) of deracemization for (S)-**1**.

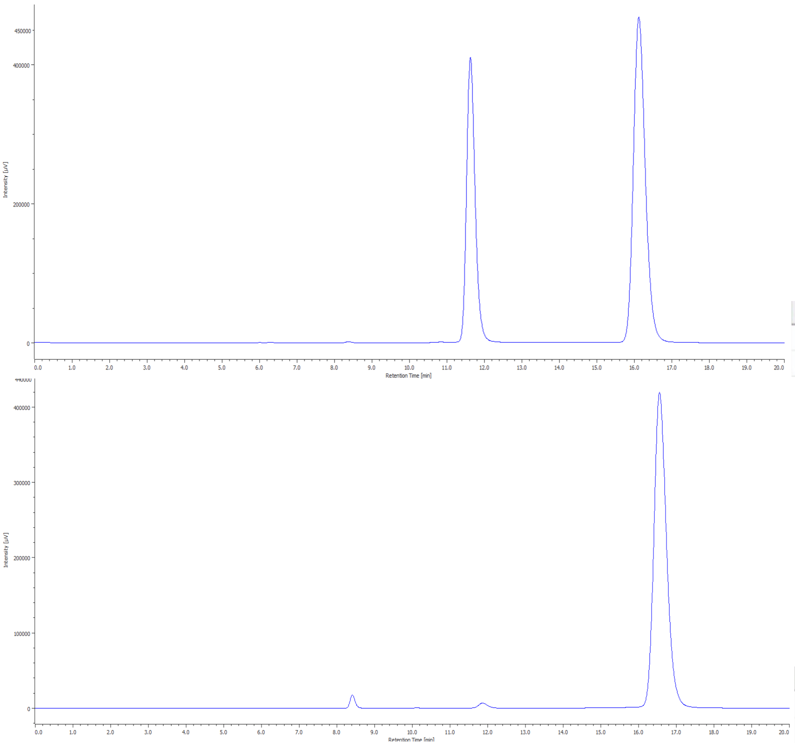

tR	Area	Height	Area%	Height%
11.617	6489220	409524	38.665	46.703
16.108	10293679	467340	61.333	53.297

tR	Area	Height	Area%	Height%
11.858	110804	6609	1.264	1.619
16.550	8648680	401719	98.658	98.381

Figure S9: Start (top) and end (bottom) of deracemization for (R)-**2**.

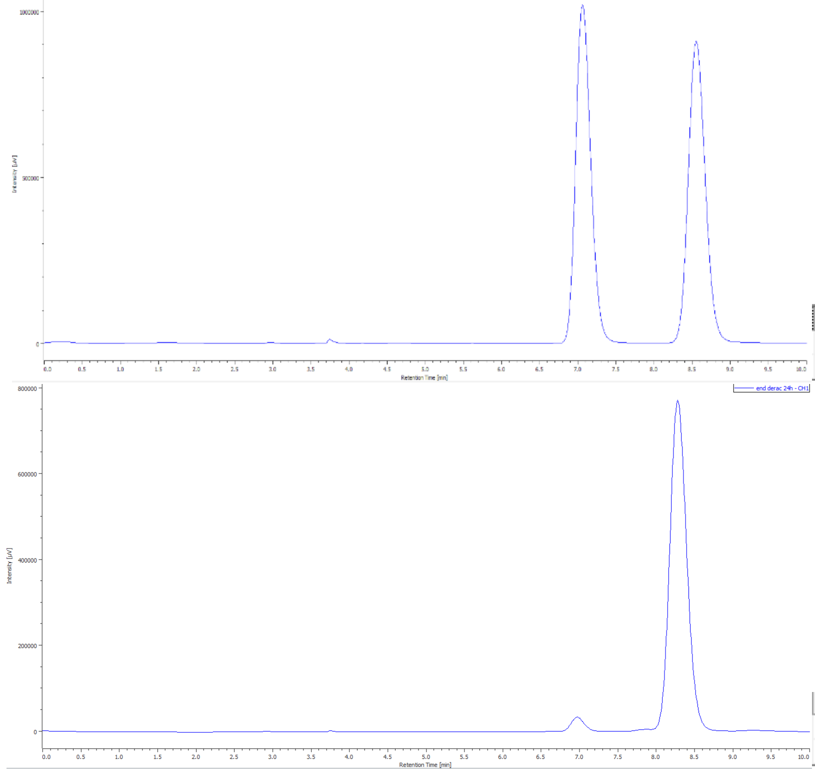

tR	Area	Height	Area%	Height%
7.058	14007614	1016940	49.978	52.861
8.550	14020020	906852	50.022	47.139

tR	Area	Height	Area%	Height%
6.967	156966	19818	1.316	2.514
8.275	11766069	768384	98.684	97.486

Figure S10: Start (top) and end (bottom) of deracemization for (R)-**3**

6. Electrochemically assisted racemization optimization

For each table, the enantiomeric excess was calculated using chiral-phase HPLC according to the method detailed in section 2.

Table S1: Initial additive screening.

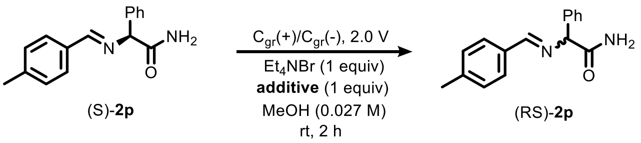

Entry	**Additive**	ee t=0 h (%)	ee t=1 h (%)	ee t=2 h (%)
1	Et_4NI		-	54
2	Pivalic acid		100	100
3	Formic acid		100	100
4	Et_3N		98	89
5	(-)-limonene		28	4
6	(+/-)-limonene		31	3
7	γ-terpinene	100	17	2
8	Linalool		24	2
9	Ocimene		21	3
10	Myrcene		20	2
11	α-terpinene		65	19
12	Cyclohexene		21	3
13	Cyclohexane		100	98
14	Toluene		100	98

Table S2: Substrate screening.

(S)-**X** —[$C_{gr}(+)/C_{gr}(-)$, 2.0 V; Et_4NBr (1 equiv); cyclohexene (1 equiv); MeOH (0.027 M); rt, 2 h]→ (RS)-**X**

Entry	**Substrate**	ee t=0 h (%)	ee t=1 h (%)
15	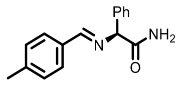 (S)-**2p**		21
16	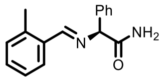 (S)-**2**	100	0
17	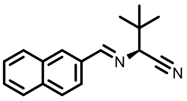 (S)-**1**		0

Table S3: Electrode material screening.

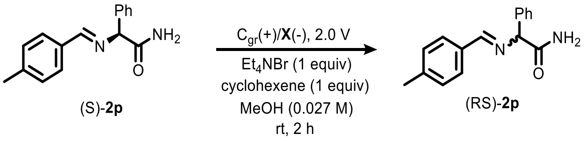

Entry	**Cathode material**	ee t=0 h (%)	ee t=1 h (%)
18	C_{gt}		21
19	Stainless steel	100	72
20	Nickel		13

Table S4: Supporting electrolyte screening.

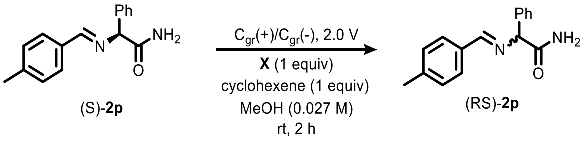

Entry	**Supporting electrolyte**	ee t=0 h (%)	ee t=1 h (%)
21	Et_4NBr		21
22	Et_4NI	100	54
23	Et_4NOTs		40

Table S5: Solvent screening.

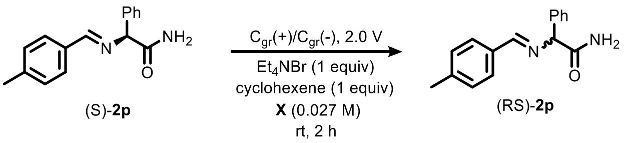

Entry	**Solvent**	ee t=0 h (%)	ee t=2 h (%)
24	MeOH	100	2
25	MeCN		71

Table S6: Substrate concentration screening.

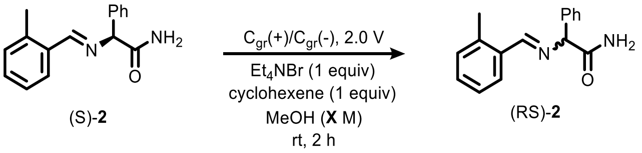

Entry	**Substrate concentration (M)**	ee t=0 h (%)	ee t=1 h (%)
26	0.027	100	4
27	1.07		15

Control experiments

Table S7: control experiments for the electrochemically assisted racemization.

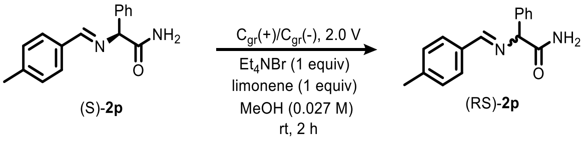

Entry	**Change to the original conditions**	ee t=0 h (%)	ee t=2 h (%)
28	No current	100	95
29	No current and MeONa (6 equiv)		27[a]
30	No limonene		91
30	No light		2
31	No air		2

Table S8: control experiment for degradation of the substrate.

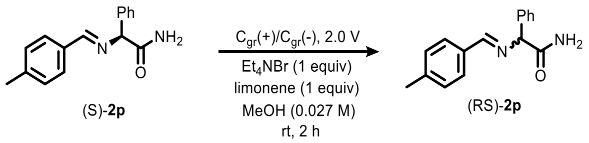

Entry	**Current (mA)**	Substrate t=0 h (%)	Substrate t=2 h (%)	Substrate t=24 h (%)
32	10	100	100	85
33	No			80

The percentage of substrate was calculated by quantitative ^{1}H NMR using trichloroethylene as external standard.

7. Mechanistic investigation

Hydrogen headspace measurement

To a 10 mL vial equipped with a stirring bar and 2 graphite electrodes, (S)-**1** (50 mg, 0.2 mmol, 1 equiv), Et_4NBr (158 mg, 0.2 mmol, 1 equiv) and cyclohexene (19 µL, 0.2 mmol, 1 equiv) were added in 7.5 mL MeOH. The solution was stirred until homogeneous and electrolyzed for 2h setting the cell potential to 2.0V. After the reaction, 5 mL of the headspace volume was removed for GC analysis. Then, 0.5 mL were sampled from the mixture and quenched in 0.15 mL of a NH_4Cl solution in EtOH (0.1 M). The final composition and enantiomeric excess in the liquid phase ($e.e._{liq}$) were determined by chiral HPLC (see section 2). Subsequently, the graphite SK-50 electrodes were washed with MeOH (3 × 20 mL) and polished using 1 and 0.3 µm alumina powder.

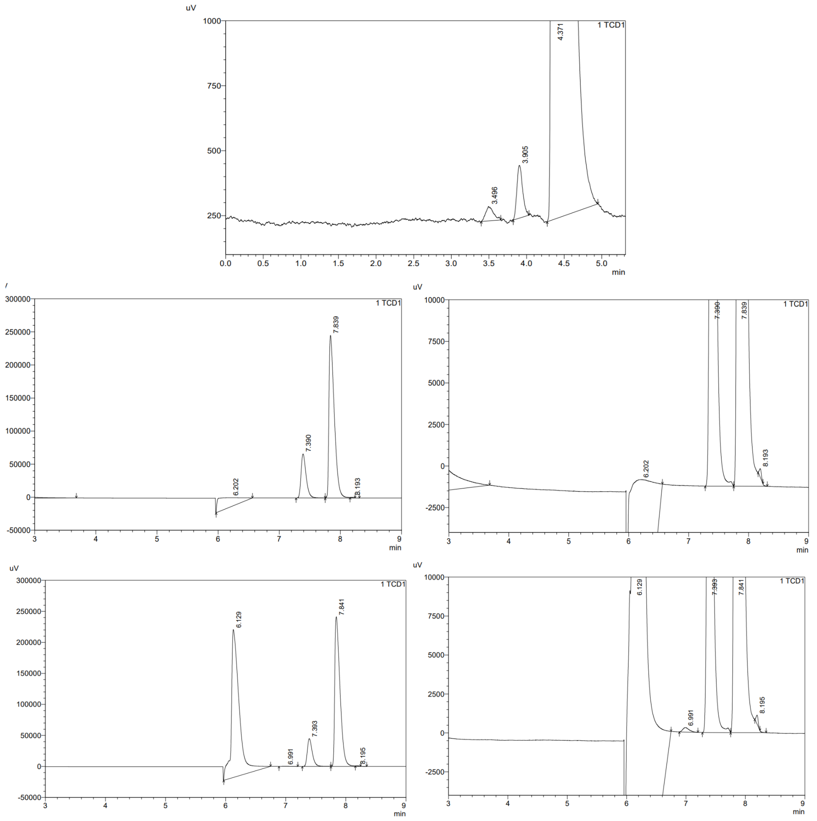

Figure S11: GC-TCD chromatogram of a synthetic mixture of 1% H_2 and 99% N_2 without using bypass, therefore offset of 3.45 minutes less, see table S9 for corrected retention times (top). before electrolysis before and after zoom (middle left and right), after 2 h of electrolysis and zoom (bottom left and right).

Table S9: Identification of gases according to their retention time.

**Retention time (min)**	**Identification**
6.129	CO_2
6.991	H_2
7.393	O_2
7.841	N_2

Additive degradation

To a 10 mL vial equipped with a stirring bar and 2 graphite electrodes, (S)-**1** (57 mg, 0.23 mmol, 1 equiv.), Et_4NBr (182 mg, 0.23 mmol, 1 equiv.) and cyclohexene (22 µL, 0.23 mmol, 1 equiv.) were added in 8.5 mL MeOD-d_4. The solution was stirred until homogeneous and electrolyzed for 2h setting the cell potential to 2.0V. After the reaction, 5 mL of the headspace was removed for GC analysis. Then, 0.5 mL were sampled from the mixture and quenched in 0.15 mL of a NH_4Cl solution in EtOH (0.1M). The final composition and enantiomeric excess in the liquid phase (e.e.$_{liq}$) were determined by chiral-phase HPLC. Subsequently, the graphite SK-50 electrodes were washed with MeOH (3 × 20 mL) and polished using 1 and 0.3 µm alumina powder.

Table S10: Additive degradation analysis.

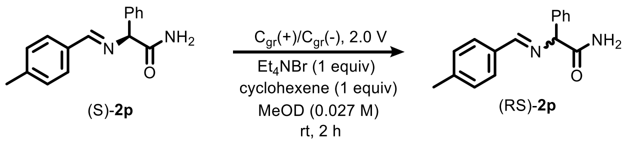

Entry	**Current**	Cyclohexene t=0 h (%)	Cyclohexene t=1 h (%)	Cyclohexene t=3 h (%)
34	No	100	100	n/a
	Yes	n/a	n/a	87

The percentage of cyclohexene was calculated by ^1H NMR by ratio with the substrate peaks.

GC-MS of the reaction mixture

To a 10 mL vial equipped with a stirring bar and 2 graphite electrodes, (S)-**1** (50 mg, 0.2 mmol, 1 equiv), Et_4NBr (158 mg, 0.2 mmol, 1 equiv.) and cyclohexene (19 µL, 0.2 mmol, 1 equiv) were added in 7.5 mL MeOH. The solution was stirred until homogeneous and electrolyzed for 2 h setting the cell potential to 2.0 V. Then, 0.5 mL were sampled from the mixture and quenched in 0.15 mL of a NH_4Cl solution in EtOH (0.1M) for composition and enantiomeric excess measurement in the liquid phase (e.e.$_{liq}$) by chiral-phase HPLC (see section 2 for methodology). Another 1 mL was sampled from the mixture to which 1 mL of ethyl acetate was added and submitted to a GC-MS analysis. Subsequently, the graphite SK-50 electrodes were washed with MeOH (3 × 20 mL) and polished using 1 and 0.3 µm alumina powder.

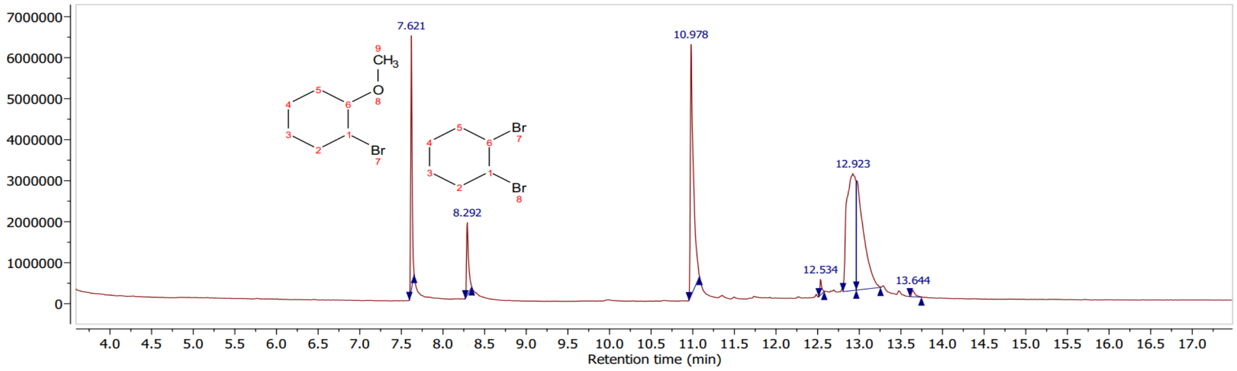

Figure S12: Chromatograms of the reaction mixture measured after 2 hours of electrolysis.

Cyclic voltammetry study

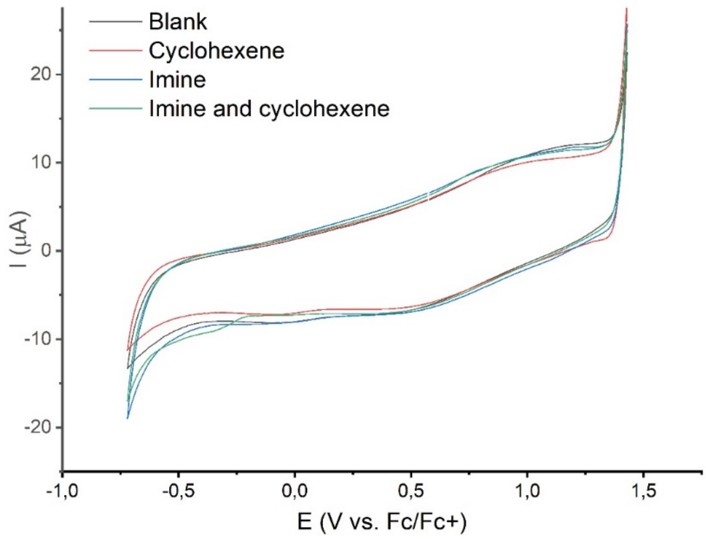

Figure S13: Cyclic voltammograms recorded at a glassy carbon disk at 100 mV·s^{-1} in 0.1M Et_4NBr /MeOH. For all the CVs the potential was cycled in positive direction first. black line: blank; orange line: cyclohexene (3 mM); blue line: imine (3 mM); green line: Phenyl glycine and cyclohexene (3 mM both). The cyclic voltammogram does not show features that can be related to the details of the mechanism. The oxidation of the cyclohexene might likely happen beyond the oxidation of the solvent.

pH control test

Racemization of the (RS)-**1** was performed as described in the general procedure section. pH was tracked over time every minute for 10 minutes, after 1hour and after 24 hours by taking 0.05 mL of the reaction mixture and adding 0.45 mL pH indicator (Hach Wide Range pH 4-12 Indicator Solution).
As a control experiment, racemization reaction was performed as described in the general procedure without cyclohexene and pH was measured as described above after 1 hour and 24 hours as and an HPLC measurement has been taken.

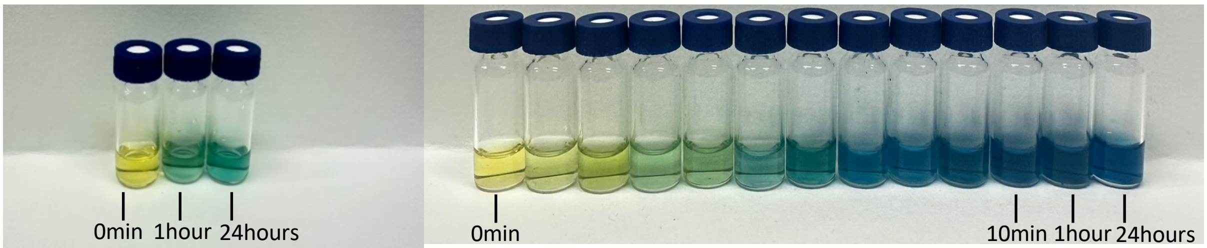

Figure S14: On the left, control experiment without cyclohexene with pH ranging from 6 to 9 (0, 1 hour and 24 hours). On the right, racemization experiment with pH ranging from 6 to 12 (0-10 minutes, 1 and 24 hours).

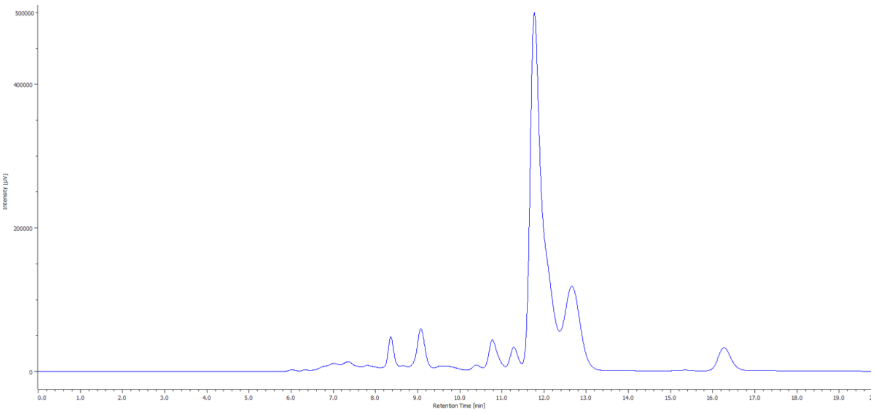

Figure S15: Control experiment, attempt at racemization of phenylglycine derivative (S)-**2** in the absence of cyclohexene showing degradation of the phenylglycine derivative (S)-**2**.

8. Determination of solubility via Crystal 16

(RS)-**1** and **(**RS)-**2** solubilities were evaluated using a Crystal 16. For both compounds, 8 different concentrations were prepared in methanol to build a solubility curve**. Three heating and cooling cycles from 0 to 60 °C were performed with a rate of 0.5 °C/min and under 1000 rpm.**

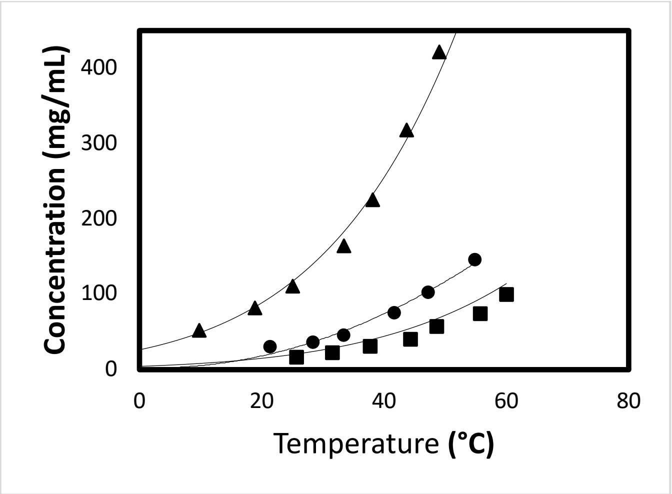

Figure S16: Solubility curve of (RS)-**1** with triangles, **(**RS)-**2** with triangles and (RS)-**3** with circles**.**

9. ^{1}H NMR

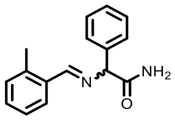

(RS)-2-((2-methylbenzylidene)amino)-2-phenylacetamide [(RS)-2]. The product was obtained as a white powder. ^{1}H NMR (300 MHz, $CDCl_3$) δ 8.27 (s, 1H), 7.70 (d, *J* = 8.3 Hz, 2H), 7.48 (dd, *J* = 6.6, 1.7 Hz, 2H), 7.41 – 7.20 (m, 5H), 4.97 (s, 1H), 2.40 (s, 3H). ^{1}H NMR (300 MHz, MeOD) δ 7.17 (s, 1H), 6.43 (d, *J* = 7.6 Hz, 1H), 5.95 (d, *J* = 7.0 Hz, 2H), 5.88 – 5.44 (m, 6H), 3.47 (s, 1H), 0.97 (s, 3H).

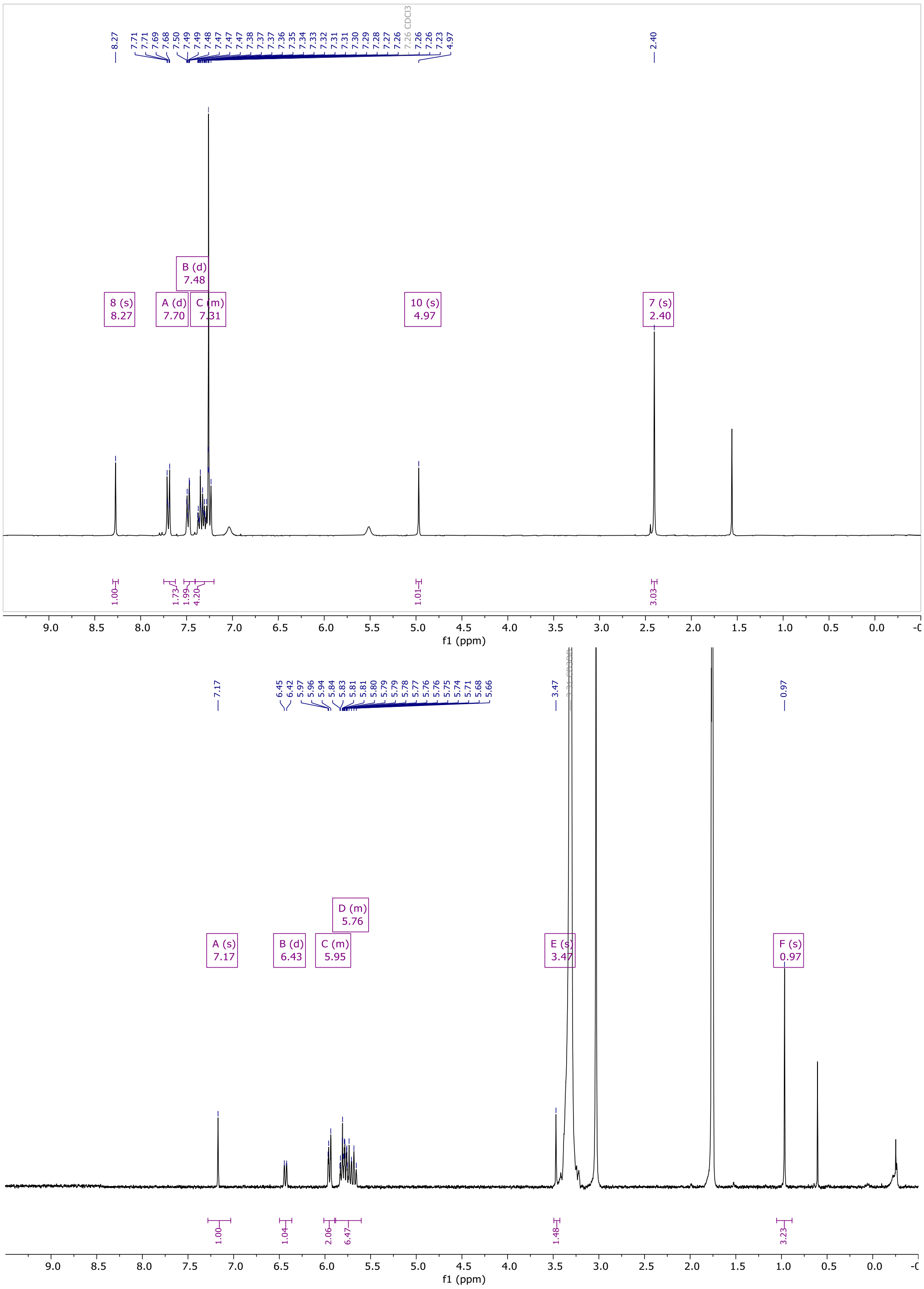

Figure S17: ^{1}H NMR of (RS)-**2** in $CDCl_3$ (top) and MeOD (bottom).

10. Flow chemistry

Table S11: Control experiment for degradation of the substrate.

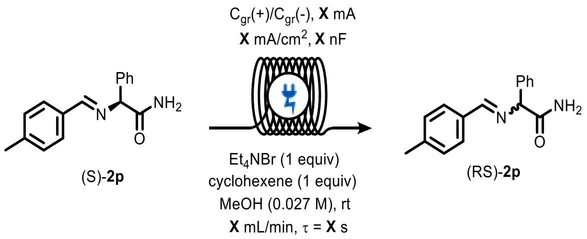

Entry	**Current (mA)**	Flow rate (mL/min)	Residence time (s)	Electrolysis time for 7.5 mL (min)	ee after electrolysis (%)
26	10	n/a	n/a	120	0
35	44	1.1	38	6.8	100
36	88	2.2	19	3.4	68
37	88	0.55	76	13.6	56*

**Partial degradation of the starting material.*

11. References

1. I. Baglai, M. Leeman, K. Wurst, B. Kaptein, R.M. Kellogg, and W.L. Noorduin, *Chem.Com.*, **2018**, **54**,10832-10834.

2. W. L. Noorduin, T. Izumi, A. Millemaggi, M. Leeman, H. Meekes, W. J. P. Van Enckevort, R. M. Kellogg, B. Kaptein, E. Vlieg and D. G. Blackmond, *J. Amer. Chem. Soc.,* 2008, ***130***, 1158–1159.

3. C. Lopes, Y. Cartigny, C. Brandel, V. Dupray, C. Body, O. Shemchuk, T. Leyssens, (2023). *Chem. Eur. J.*, ***29***, 35.

4. M. Regnier, C. Vega, D. I. Ioannou and T. Noël, *Chem. Soc. Rev.*, 2024, **53**, 10741–10760.
